# Molecular movement in the *Arabidopsis thaliana* female gametophyte

**DOI:** 10.1007/s00497-017-0304-3

**Published:** 2017-07-10

**Authors:** Robert M. Erdmann, Anja Hoffmann, Heidi-Kristin Walter, Hans-Achim Wagenknecht, Rita Groß-Hardt, Mary Gehring

**Affiliations:** 1 Whitehead Institute for Biomedical Research, Cambridge, MA 02142 USA; 2 Department of Biology, Massachusetts Institute of Technology, Cambridge, MA 02139 USA; 3 Department of Plant Molecular Genetics, University of Bremen, 28359 Bremen, Germany; 4 Institute for Organic Chemistry, Karlsruhe Institute of Technology, 76131 Karlsruhe, Germany

**Keywords:** Female gametophyte, Central cell, Egg apparatus, Symplastic movement, Small RNAs, *Arabidopsis thaliana*

## Abstract

*****Key message***:**

**Size limits on molecular movement among female gametes.**

**Abstract:**

Cellular decisions can be influenced by information communicated from neighboring cells. Communication can occur via signaling or through the direct transfer of molecules. Movement of RNAs and proteins has frequently been observed among symplastically connected plant cells. In flowering plants, the female gametes, the egg cell and central cell, are closely apposed within the female gametophyte. Here we investigated the ability of fluorescently labeled dyes and small RNAs to move from the *Arabidopsis thaliana* central cell to the egg apparatus following microinjection. These results define a size limit of at least 20 kDa for symplastic movement between the two gametes, somewhat larger than that previously observed in *Torenia fournieri*. Our results indicate that symplastic connectivity in *Arabidopsis thaliana* changes after fertilization and suggest that prior to fertilization mechanisms are in place to facilitate small RNA movement from the central cell to the egg cell and synergids.

## Introduction

The female gametophyte is the site of fertilization in flowering plants. As is typical of most angiosperms, the *Arabidopsis thaliana* female gametophyte consists of two haploid synergid cells, a haploid egg cell, a diploid central cell, and three haploid antipodal cells. The nuclei of the female gametophyte arise from mitotic division of a single functional megaspore and occupy a common cytoplasm until cellularization commences shortly after the last division (Drews and Yadegari 2004). Despite this, female gametophytic cells have very different fates. The antipodal cells undergo degeneration around the time of fertilization, the synergid cells attract the pollen tube, the egg cell is fertilized by a haploid sperm to develop into the diploid embryo, and the central cell is fertilized by the second haploid sperm to develop into triploid endosperm tissue. Maintenance of gametophytic cell fate is closely tied to position along the ovule micropylar–chalazal axis, but can be altered, for example, in mutants predicted to affect auxin biosynthesis or RNA splicing (Tekleyohans et al. 2017). Several pieces of evidence point to communication among cells of the female gametophyte—genes expressed in one cell can influence the fate of other cells (Kägi et al. 2010; Völz et al. 2012; Krohn et al. 2012; Wu et al. 2012). It is thus important to understand the potential for physical cell-to-cell molecular movement to mediate this communication. Molecular movement between the central cell and egg apparatus (the egg cell and synergids) has been most extensively analyzed in *Torenia fournieri*, a species in which half of the embryo sac extrudes from the ovule, rendering it physically accessible for manipulation (Han et al. 2000). These studies have shown that the central cell and egg cell are symplastically connected before fertilization, but that permeability decreases substantially as the female gametophyte matures, and is eliminated at fertilization. Despite the importance of *A. thaliana* as a model genetic system for understanding female gametophyte development and cell fate specification, there has been little investigation of molecular movement within the female gametophyte. Like in other angiosperms, there is no cell wall between the *A. thaliana* egg and central cell (Mansfield and Briarty 1991), suggesting reduced barriers to movement compared to other plant tissues. We previously developed a microinjection protocol for *A. thaliana* that allowed us to microinject the central cell of living ovules (Völz et al. 2013). Here, we used this experimental system to analyze the ability of larger macromolecules, including fluorescent tracers and labeled 24 nucleotide small RNAs (with two dyes as an energy transfer pair, “RNA traffic lights” (Walter et al. 2017; Arndt et al. 2016; Holzhauser and Wagenknecht 2013)) to move from the central cell to the egg apparatus before fertilization and from the primary endosperm to the zygote after fertilization.

## Materials and methods

### Plant material and growth conditions

Plants were grown on soil in growth chambers under long-day conditions (16 h light and 8 h dark) at approximately 18 °C. All experiments were performed on wild-type L*er* plants or FGR7.0 L*er* plants (Völz et al. 2013). The largest closed flower buds were emasculated and then pollinated after 1–2 days.

### Ovule and seed microinjection

Ovules/seeds were removed from pistils/siliques while maintaining attachment to the placenta. Tissue was mounted on pads of C + media (4 mM CaCl_2_, 1 mM MgSO_4_, 14.5% sucrose, 3% Polyethylene glycol 4000, 0.01% H_3_BO_3_, and MilliQ H_2_O), surrounded by C- media (C + media with 2% alginic acid in place of CaCl_2_) and then covered with halocarbon oil. Injections were performed as previously described (Völz et al. 2013). A piezo-stepper (PMZ 20, Frankenberger, Filching, Germany) and a FemtoJet (Eppendorf) were used in tandem to perform the pressure microinjections under an inverted epifluorescence microscope (Leica CTR 6000). The approximate concentration of RNA A or RNA B in microinjection solution was 1 ng/µL.

### Microscopy

Confocal laser scanning microscopy was performed on an LSM 780 (Zeiss) with the following conditions: FITC excitation: 488 nm, FITC emission: 499–525 nm; rhodamine excitation: 561 nm, rhodamine emission: 570–695 nm; RNA acceptor excitation: 561 nm, RNA acceptor emission: 606–695 nm; RNA donor excitation: 458 nm, RNA donor emission: 472–543 nm. Fluorescent tracer imaging occurred between 5 and 95 min post-injection. sRNA injection imaging occurred between 30 min and 4 h post-injection. Determinations of molecular movement were made blind by two authors.

### Image manipulation

**Fig. 1:**
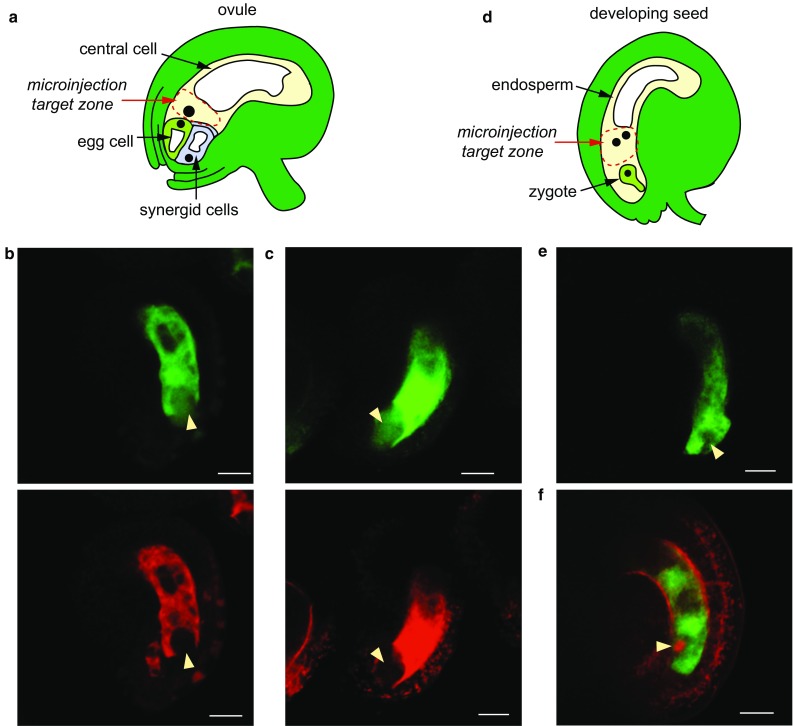
Assaying passive dye permeability in the female gametophyte before and after fertilization. **a** Schematic of Arabidopsis ovule. **b** Pre-fertilization co-injection of L*er* with 10 kDa FITC (*green*) and 70 kDa rhodamine (*red*). Image captured 45 min following injection. **c** Pre-fertilization co-injection of L*er* with 20 kDa FITC (*green*) and 70 kDa rhodamine. Image captured 80 min following injection. *Yellow arrowheads* indicate the position of the egg apparatus (the cluster of the egg cell and synergid cells). **d** Schematic of the developing seed, about 18 h after pollination. **e** Post-fertilization injection of L*er* with 10 kDa FITC. Image captured 90 min after injection. **f** Post-fertilization injection of L*er* FGR7.0 with 10 kDa FITC. RFP (*red*) is expressed within the zygote. Image captured 60 min after injection. *Yellow arrowheads* mark the position of the zygote. All scale bars, 20 μm

**Fig. 2:**
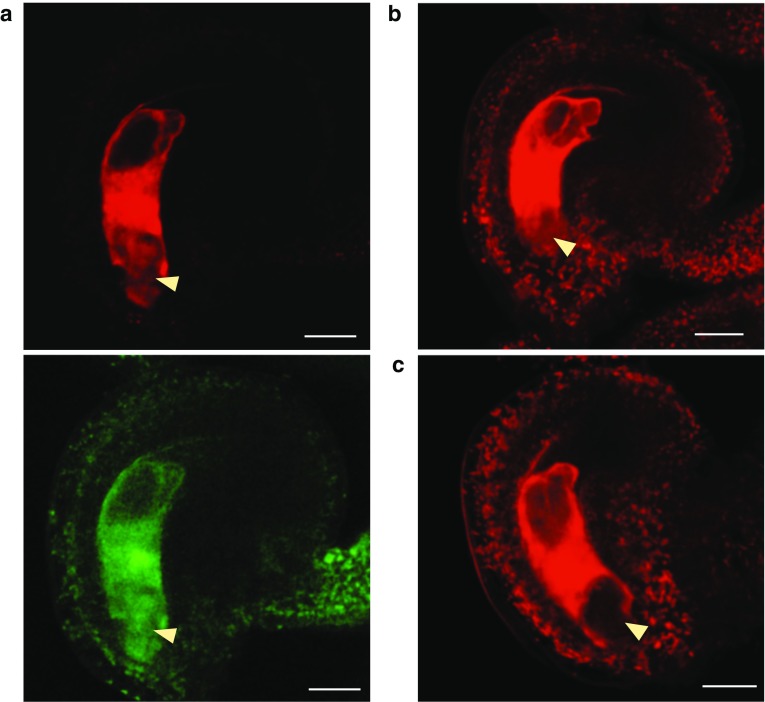
Assaying small RNA movement between the cells of the female gametophyte using a FRET system **a** Pre-fertilization injection of 24 nucleotide RNA “A.” Total sRNA signal shown in *red*, single-stranded signal shown is *green*. Movement is observed. **b** Pre-fertilization injection of 24 nucleotide RNA “B,” showing total sRNA signal. Image captured 60 min following injection. Movement is observed. **c** Pre-fertilization injection of 24-nucleotide RNA “B,” showing total sRNA signal. Image captured 65 min following injection. No movement is observed. *Yellow arrowheads* indicate the position of the egg apparatus. *Scale bars*, 20 μm

Image intensities were scaled to the maximum signal level found in each channel. Brightness adjustments were performed identically for every channel within an individual image. All images in Figs. 1 and 2 represent a single focal plane. FIJI (Schindelin et al. 2012) was used to apply a Gaussian filter (radius of 2.0 pixels) to each micrograph.

### Small RNA sequences and construction

Labeled small RNAs were synthesized in the manner of Walter et al. (2017).

- RNA “A”:
- 5′ (PO_3_^2−^A)AA GAC AAU GAC AAA (U^)UC UUG GC(G-OMe) 3′
- 3′ (OMe-C)A UUU CUG UUA CUG UU(U*) AAG AAC (C-PO_3_^2−^) 5′
- RNA “B”:
- 5′ (PO_3_^2−^U)AU AUG CAA GUC CGG CCA (U^)AC AG(C-OMe) 3′
- 3′ (OMe-C)U AUA UAC GUU CAG GCC GG(U*) AUG (U-PO_3_^2−^) 5′
- Positions marked with * were conjugated to Label 1.
- Positions marked with ^ were conjugated to Label 2.
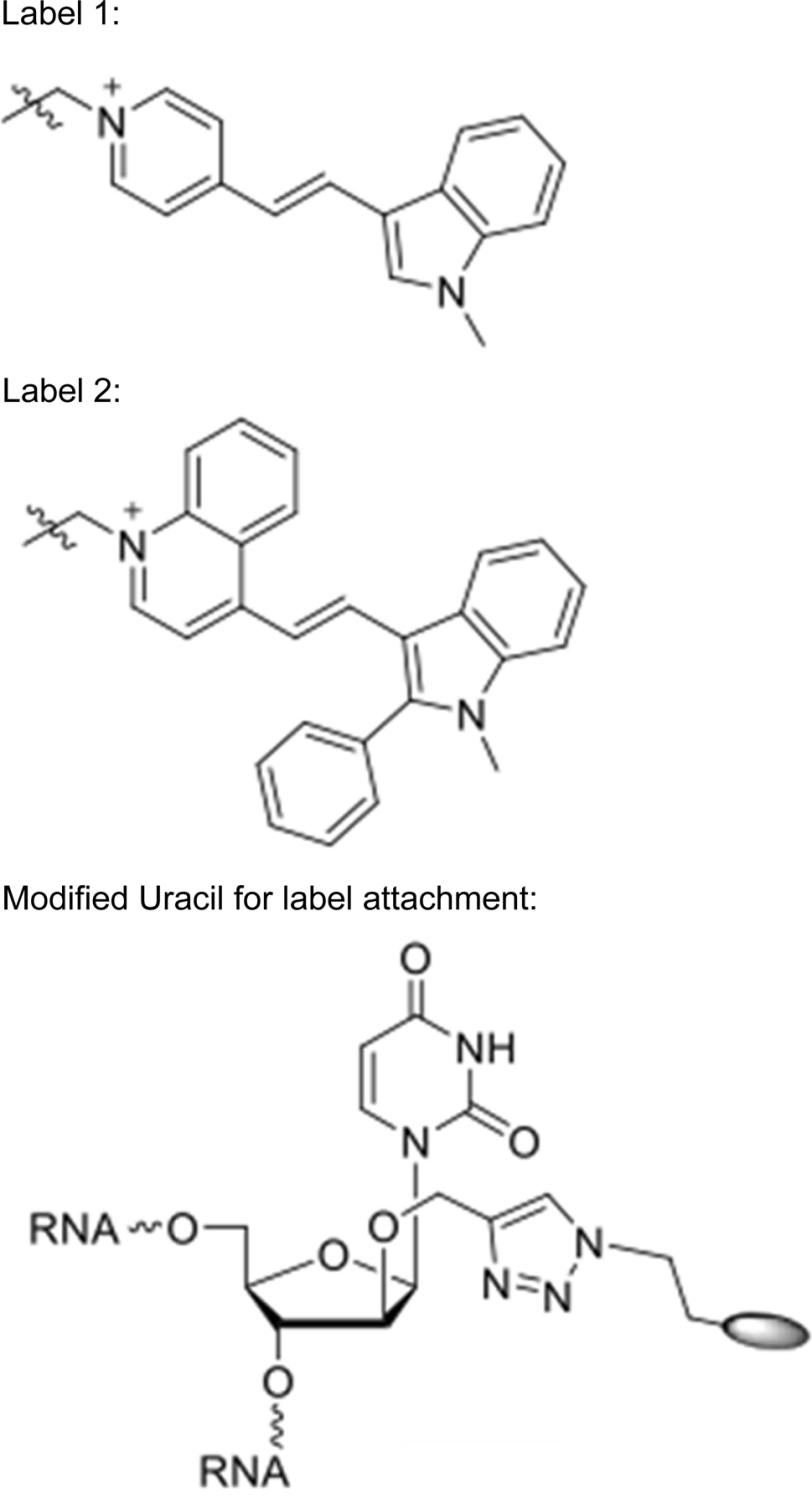

## Results

### Limits on passive dye diffusion in the female gametophyte

We measured the ability of fluorescent dyes ranging in size from 805 Da to 70 kDa to diffuse from the central cell to the egg apparatus before fertilization. L*er* flowers were emasculated and allowed to mature for 1–2 days. Ovules were removed from pistils while still connected to the placenta and embedded on media plates that allowed for maintenance of ovule viability over the course of the experiment (Völz et al. 2013). Smaller fluorescent FITC dyes (green) were coinjected into the central cell along with 70 kDa rhodamine dye (red). At 1 and 2 days after emasculation (DAE), 805 Da, 10 kDa, and 20 kDa dyes entered the egg cell within 10 min of injection into the central cell (Table 1 and Fig. 1). Z-stack imagery of the entire depth of the female gametophyte indicated that the dye signal was found both within the egg cell and synergid cells (data not shown). By contrast, in 35 injections performed 1–2 DAE, movement of the 70 kDa dye out of the central cell was not observed (Table 1). These results indicate that the molecular limit on passive movement in the mature *A. thaliana* female gametophyte is between 20 and 70 kDa.

**Table 1:** Assessment of fluorescent dye movement pre- and post-fertilization

Tracer size	From CC to egg 1 DAE^a^ (moved/total)	From CC to egg 2 DAE (moved/total)	From primary endosperm to zygote 18 HAP^b^ (moved/total)
805 Da FITC	6/6	–	–
10 kDa FITC	7/7	13/13	0/9
20 kDa FITC	2/2	9/9	0/6
70 kDa rhodamine	0/15	0/20	0/11

CC = central cell

^a^days after emasculation; ^b^ hours after pollination

### Passive dye diffusion is restricted after fertilization

We tested whether fertilization altered the ability of dyes to diffuse from the 1–2 cell endosperm to the zygote (Table 1 and Fig. 1). Most injections were performed approximately 18 h after pollination (HAP), by which point it was clear that fertilization had occurred throughout the silique. In contrast to the results obtained before fertilization, we were unable to detect movement of 10 or 20 kDa dyes out of the endosperm. Lack of movement was apparent in wild-type ovules (Fig. 1e) and in the FGR7.0 line (Fig. 1f), in which the egg cell was labeled with RFP. These results suggest that fertilization is associated with rapid restriction of symplastic connections between the central cell and egg cell.

### Labeled small RNAs move from central cell to egg cell

With the approximate diffusion limits assayed, we next tested the ability of fluorescently labeled sRNAs to move from the central cell to the egg apparatus. We independently injected two double-stranded RNA sequences into the central cell: RNA “A” corresponded to a 24 nt small RNA highly expressed within the developing seed (Pignatta et al. 2014), whereas RNA “B” corresponded to a 24 nt sequence not found within the *A. thaliana* genome. The sRNAs were labeled for use in fluorescence resonance energy transfer (FRET) experiments to distinguish single- and double-stranded molecules. In one microscopy channel (red), the acceptor dye (label 2) was excited directly and the same acceptor dye’s emission was monitored, providing an additive measure of both single- and double-stranded sRNA. The other channel (green) monitored the fluorescence of the donor fluorophore that was not transferred to the acceptor following excitation of the donor, thus denoting single-stranded sRNAs. sRNA duplexes are estimated to be about 16 kDa in size, with the dye pair contributing slightly less than 1 kDa. The stability of sRNAs post-injection and the photochemical stability of the applied dyes (Bohländer and Wagenknecht 2015; Walter et al. 2015) was seemingly quite high, with the signal remaining detectable at least four hours (the longest period tested) after microinjection into the central cell. In 10 of 11 microinjections, RNA “A” could be detected in the egg cell after injection into the central cell (Fig. 2a). Signal from both single and double-stranded RNAs was observed in the egg cell. By contrast, results from RNA “B” were equivocal—movement from central cell to egg cell was observed in only 4 of 8 microinjections (Fig. 2b, c).

## Discussion

These results indicate that the central cell and egg apparatus remain symplastically connected even after cellularization and suggest that multiple types of macromolecules may have the ability to be passively shared among cells of the *A. thaliana* female gametophyte. Our findings also reveal differences between *A. thaliana* and *Torenia*, the species in which these questions have been most closely examined. Unlike in *Torenia* (Han et al. 2000), we did not observe a decrease in the ability of molecules to move between cells as the female gametophyte matured (Table 1, compare 1 and 2 DAE). Additionally, the size of molecules that diffuse between the central cell and egg cell is larger in *A. thaliana* than in *Torenia*. At maturity, molecules of 3 kDa could move from the central cell to egg cell in *Torenia* about half the time, whereas earlier in development movement of 10 kDa FITC was observed (Han et al. 2000). Our studies at 2 DAE, when the female gametophyte is mature, indicate that tracers up to 20 kDa can freely pass between the cells. The size range for movement in the female gametophyte is thus similar to that observed in Arabidopsis embryos, young seedlings, and the seed outer integuments (Kim et al. 2005; Stadler et al. 2005). Analogous to findings from *Torenia* (Han et al. 2000), our data indicate symplastic isolation between the zygote and endosperm shortly after fertilization, suggesting that gamete fusion triggers modulation of the egg and or central cell extracellular matrix. Symplastic isolation has also been observed at later stages of Arabidopsis embryo and endosperm development (Stadler et al. 2005; Ingram 2010). It is not yet clear whether embryo–endosperm isolation is a common theme in flowering plants, as suspensor and endosperm share plasmodesmata in Crassulaceae (Kozieradzka-Kiszkurno and Plancho 2012), indicating the potential for molecular movement between these compartments.

Finally, we also detected movement of labeled small RNAs before fertilization. In *Torenia,* FITC-labeled morpholino 25 nt antisense oligomers injected into the central cells moved to and functioned in the synergid cells (Okuda et al. 2009). Our findings suggest that similar knockdown experiments are possible in *A. thaliana.* Additionally, this system may also facilitate in understanding properties of endogenous small RNAs.

### **Author contribution statement**

RME, RG, and MG conceived and designed experiments. RME and AH conducted experiments and analyzed data. HKW and HAW contributed new reagents. RME, RG, and MG wrote the manuscript.
